# Individual-level needle and syringe coverage in Melbourne, Australia: a longitudinal, descriptive analysis

**DOI:** 10.1186/s12913-016-1668-z

**Published:** 2016-08-19

**Authors:** Daniel O’Keefe, Nick Scott, Campbell Aitken, Paul Dietze

**Affiliations:** 1 Centre for Population Health, Burnet Institute, 85 Commercial Rd, Melbourne, VIC 3004 Australia; 2 School of Public Health and Preventive Medicine, Monash University, 99 Commercial Rd, Melbourne, VIC 3004 Australia

**Keywords:** Injecting drug use, Syringe coverage, Harm reduction, Longitudinal analysis

## Abstract

**Background:**

Coverage is used as one indicator of needle and syringe program (NSP) effectiveness. At the individual level, coverage is typically defined as an estimate of the proportion of a person who injects drugs’ (PWID) injecting episodes that utilise a sterile syringe. In this paper, we explore levels of individual syringe coverage and its changes over time.

**Methods:**

Data were extracted from 1889 interviews involving 502 participants drawn from the Melbourne drug user cohort study (MIX).

We asked questions relating to participants syringe acquisition, distribution and injecting frequency within the two weeks before interview. We created a dichotomous coverage variable that classified participants as sufficiently (≥100 %) covered if all their injecting episodes utilised at least one sterile syringe, and insufficiently (<100 %) covered if not. We categorised participants as “consistently covered” if they were sufficiently covered across interviews; as “consistently uncovered” if they were insufficiently covered across interviews; and “inconsistently covered” if they oscillated between coverage states.

Chi-square statistics tested proportions of insufficient coverage across sub-groups using broad demographic, drug use and service utilisation domains. Logistic regression tested predictors of insufficient coverage and inconsistently covered categorisation.

**Results:**

Across the sample, levels of insufficient coverage were substantial (between 22–36 % at each interview wave). The majority (50 %) were consistently covered across interviews, though many (45 %) were inconsistently covered.

We found strong statistical associations between insufficient coverage and current hepatitis C virus (HCV) infection (RNA+). Current prescription of opioid substitution therapy (OST) and using NSPs as the main source of syringe acquisition were protective against insufficient coverage.

**Conclusion:**

Insufficient coverage across the sample was substantial and mainly driven by those who oscillated between states of coverage, suggesting the presence of temporal factors. We recommend a general expansion of NSP services and OST prescription to encourage increases in syringe coverage.

**Electronic supplementary material:**

The online version of this article (doi:10.1186/s12913-016-1668-z) contains supplementary material, which is available to authorized users.

## Background

The coverage of a public health program can be defined as the extent to which it reaches its intended population [1]. It is an indicator of the effectiveness of public health interventions in reducing public health risks.

Needle and syringe programs (NSPs) seek to avert blood-borne virus (BBV) spread amongst people who inject drugs (PWID) via the distribution of sterile needles and syringes (hereafter referred to as syringe/s). The coverage achieved by NSPs at the population level refers to the proportion of PWID reached by services. At the individual level, coverage is typically defined as the proportion of a PWID’s injecting episodes that utilise a sterile syringe [2].

The sharing of used syringes is a significant contributor to the transmission of BBVs amongst PWID [3, 4]. It is estimated that globally, only 1–4 syringes are distributed per PWID per month [5], well below the World Health Organization (WHO) recommended rate of 200 syringes per PWID per year [6].

Syringe coverage is mediated by context. Service management and funding [7], dispensation policy [8], intensive policing practices [1, 9], cohesiveness of PWID networks [2], spatial service access [10], and individual demographics [11, 12] influence the ability of individuals to attain sufficient syringes and service systems to provide sufficient coverage.

Previous research has shown that insufficient coverage at the individual level is significantly associated with high-frequency injecting and not using NSPs as a primary source of syringe acquisition [2]. Insufficient individual-level coverage has also been associated with syringe re-use and receptive/distributive syringe sharing [11–13]. Despite these findings, current understanding of the causes of insufficient coverage is poor. Most research on individual coverage has been cross-sectional and consequently unable to capture variation over time – hence Bluthenthal et al.’s call for longitudinal investigation [11]. A greater understanding of coverage over time will also provide better knowledge of the predictors of insufficient coverage and enable better interventions.

The Australian context provides the ideal setting for research on patterns of syringe coverage over time. Australia’s early and comprehensive adoption of NSPs prevented an HIV epidemic in PWID, in contrast to many other countries [14, 15]. An estimated 3000+ syringe outlets service an estimated population of 90,000 PWID [16], distributing approximately 213 syringes per PWID per year [12], in excess of WHO population-level recommendations [6]. Despite greater opportunity to acquire syringes than many of their international counterparts, an estimated 16–37 % of Australian PWID experience insufficient coverage [2, 12, 17]. Consequently, research exploring the individual and structural determinants of insufficient coverage in Australia provides important information for other settings.

In this paper we analyse six years of data from an ongoing cohort of PWID in Melbourne, Australia. We aim to:

- describe the characteristics of individuals with recent insufficient coverage (insufficient syringe acquisition to cover injecting episodes within the past two weeks) across broad demographic, drug use and service utilisation domains;
- explore how the proportion of individuals with recent insufficient coverage changes over time;
- categorise participants according to their longitudinal patterns of coverage; and
- identify exposure sub-groups independently associated with individual coverage and longitudinal coverage pattern trajectories.

## Methods

### Melbourne injecting drug user cohort study

Data are drawn from the Melbourne injecting drug user cohort study (MIX), which has been described in detail elsewhere [18]. The cohort includes PWID recruited through the original MIX recruitment phase in 2008–2010 (*n* = 688), and those rolled into the study in 2011 via past involvement in the Networks II cohort (*n* = 69) [19], resulting in 757 participants. Both MIX and Networks II sought to recruit regular injectors, and despite some demographic differences between the MIX and Networks II cohorts at the 2011 roll-in (mean age in 2011 was 29 in MIX, 35 in Networks II; 16 % in MIX were born overseas, 31 % in Networks II; 54 % were currently on OST in MIX, 62 % in Networks II), the characteristics of the cohorts at baseline (2005 for Networks II) were comparable [19–21].

Eligibility criteria for the original MIX cohort were being aged 18–30 years and reporting injecting of heroin and/or methamphetamine regularly (at least once a month in the six months prior to recruitment).

### Participant sample

As of February 2015 (dataset end), 2862 separate interviews had been collected over a maximum of seven annual interview waves per participant. As the necessary coverage questions were not introduced into the questionnaire until June 2010, all interviews prior to this date (902 interviews, 184 participants) were excluded from analysis. Furthermore, as we intended to analyse changes to coverage longitudinally, only participants with two or more interviews after June 2010 were retained, excluding a further 71 participants. This process resulted in an amended dataset of 502 participants and 1889 interviews across a maximum of six separate interview waves. Study retention was high, with 85 % of these participants having at least three interviews.

The demographic and drug use patterns of the total cohort and the amended sample used in analysis were similar, though current employment was 7 percentage points higher, and current OST prescription 21 percentage points higher, among the amended sample. Comparisons between the two sets of data at first interview are presented in Additional file 1.

### Measures

To measure syringe retention, we asked the following questions:

- *“In the last two weeks, how many new syringes in total did you get?”*
- *“In the last two weeks, how many syringes did you give away or sell to others?”*

The MIX questionnaire records past week use and injecting frequency for 18 drug types. Past week injecting frequencies for each drug type were summed to create a total injecting frequency variable.

Using a method of calculating individual syringe coverage adapted from Bluthenthal et al. [11], we subtracted the number of syringes sold or given away from the number of syringes acquired. We then multiplied past week injecting frequency by two to create a consistent time frame for the measure (rather than syringe collection being halved, as initial inspection showed less variance for injection frequency, suggesting it is the more consistent practice). We then divided the number of syringes retained by past two-week injecting frequency and then multiplied by 100, resulting in a percentage of injecting episodes that utilised a sterile syringe. The formula for individual coverage measurement was therefore:

$$ \frac{\left( syringes\  acquired- syringes\  distributed\right)}{\left( past\  week\  injecting\  frequency\ x\ 2\right)} \times 100 $$

Recent individual coverage was considered to be sufficient if every reported episode of injecting was covered by at least one reported sterile syringe, or ≥100 % individual coverage. A dichotomous variable, “recent coverage” (≥100 % coverage / <100 % coverage), was applied to each interview with valid data, classifying participants as either sufficiently or insufficiently covered for the two weeks before interview.

Coverage was only calculated for participants who reported both syringe acquisition and injecting within the two-week period (as the absence of either parameter precludes calculation). Missing data accounted for 44 % (832 observations) of all coverage responses. Of these missing data, most (602 observations, 72 % of all missing responses) resulted from injecting abstinence.

### Sub-group selection

We chose exposure variables a priori, including predictors in Bluthenthal et al.’s [11] original coverage paper and recent work by McCormack et al. [17]. Broadly, these subgroups fall within demographic, drug use characteristics and service utilisation domains. **Demographic:** “sex” (male/female), “Indigenous status” (yes/no), “WHO definition of youth” (≤24 years/>24 years); “highest level of education” (<year 10/year 10–11/year 12, higher education, trade), “weekly income” (around median: <$400/≥$400), “employment status” (employed/unemployed), “stable accommodation” (yes/no), “country of birth” (Australia/other), “arrest (past twelve months)” (yes/no). **Drug use characteristics**: “injecting career” (around median: <13 years/≥13 years), “heroin injection (past month)” (yes/no), “methamphetamine injection (past month)” (yes/no), “Hazardous drinking scale score – *derived from Audit-C scale*” (abstinent/<8 points/≥8 points) [22], “receptive syringe sharing (past month) - *derived from BBV-TRAQ-SV*” (yes/no), “injection of another person (past month) - *derived from BBV-TRAQ-SV*” (yes/no), “been injected by another person (past month) – *derived from BBV-TRAQ-SV*” (yes/no), “BBV-TRAQ-SV injecting risk scale score” (continuous measure) [23], “hepatitis C virus serology (HCV) status” (three categories: positive (RNA+)/exposed (Antibody+, RNA-)/negative (Antibody-, RNA-), “injecting more than usual in the past six months” (yes/no), “solitary injecting >80 % of the time” (yes/no). **Service utilisation:** “current opioid substitution therapy prescription (OST)” (yes/no), “NSP as usual source of syringe acquisition (past month)” (yes/no). An amended version of the MIX questionnaire, relevant to this analysis, is presented in Additional file 2: Appendix 1.

### Analysis strategy

We categorised participants with at least two instances of valid coverage data into three distinct subgroups according to longitudinal experience of the dichotomised recent coverage variable: “consistently covered” if all valid coverage data was recorded as sufficient, “consistently uncovered” if all valid coverage data was recorded as insufficient, and “inconsistently covered” if participants had at least one change between the two states of coverage across interviews.

The three coverage pattern groups were comparable in terms of missing data and attrition patterns. In the consistently covered group, 91 % of participants had three or more interviews and 27 % missing coverage data. In the consistently uncovered group, 82 % had three or more interviews and 26 % missing data. The inconsistently covered group had 92 % with three or more interviews and 22 % missing data.

### Statistical analysis

Proportional differences between participants experiencing sufficient or insufficient coverage at their first interview and their most recent interview were tested using chi-square statistics for categorical variables and Wilcoxon rank-sum testing for non-parametric continuous variables. Proportional differences between the three coverage pattern groups at first interview were tested using chi-square statistics for categorical variables and Kruskal-Wallis testing for non-parametric continuous variables.

Logistic regression was used to determine cross-sectional predictors of insufficient coverage from the dichotomous recent coverage variable. Initial inspection suggested that a binary coverage pattern variable of consistently covered/inconsistently covered be examined (placement in the inconsistently covered group as the outcome of interest), with too few cases of those consistently uncovered to allow analysis. The chosen time point of analysis was the first interview for each participant so as to minimise any bias across time due to differences in number of interviews.

Statistical significance was set at *p* < 0.05. All analyses were carried out using Stata 13.1 for Windows (StataCorp LP, TX, USA).

## Results

### Participant demographics

At first interview, the amended sample of 502 participants was predominately male (64 %), Australian-born (82 %), largely non-indigenous (95 %), unemployed (78 %) and living in stable accommodation (85 %). Mean age at first interview was 30. For those reporting injecting within the month prior to interview (*n* = 416), heroin was the most commonly injected drug (73 %), followed by methamphetamine (11 %). The remaining 16 % of participants most commonly injected either some form of OST or other pharmaceutical opioid.

### Coverage characteristics across the cohort

Participants who reported syringe acquisition in the two weeks before interview collected syringes from any source a median of two times (interquartile range (IQR): 1–3) at both first and most recent interview. Participants collected a median of 20 syringes at first and most recent interview (IQRs of 10–70 and 10-100 respectively), and gave away/sold a median of one syringe (IQR: 0–8) at first interview and zero syringes (IQR: 0–10) at most recent interview. After subtraction of distributed syringes, participants retained a median of 16 syringes at both their first and most recent interview (IQRs of 6–48 and 5–65 respectively).

For those not reporting injecting abstinence in the week before interview, median self-reported injecting frequency was five times (IQR: 2–11) at both first and most recent interview (IQR: 2–14).

Median coverage percentages at first and most recent interview were 165 % (IQR: 92–353 %) and 175 % (IQR: 100–357 %) respectively. Despite the median percentage coverage being greater than 100 %, recent insufficient coverage was substantial; 26 % and 25 % of the sample were insufficiently covered at their first and most recent interview respectively.

**Fig. 1:**
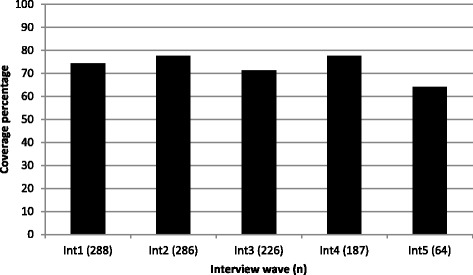
Percentages of sufficient coverage across interview waves

The percentages of participants with recent sufficient coverage across all interviews are presented in Fig. 1.

### Cross-sectional sufficient/insufficient coverage across exposure sub-groups

Proportions of sufficient and insufficient coverage were stable over time across many sub-groups. Those with insufficient coverage were more likely to report episodes of increased injecting frequency lasting ≥1 month in the past six months (*X*^2^ = 4.28, *p* = 0.039) and recent injection of methamphetamine (*X*^2^ = 15.18, *p* = <0.001). Those with sufficient coverage were more likely to report injecting careers equal to or longer than 13 years (*X*^2^ = 15.63, *p* = <0.001) and current OST prescription (*X*^2^ = 12.11, *p* = 0.001). These findings were significant at most recent interview, but not at first interview. Participants arrested within the past twelve months were more likely to report insufficient coverage at first interview (*X*^2^ = 3.91, *p* = 0.048), but not at most recent.

Insufficient coverage was also associated with risk practices. Those with insufficient coverage were more likely to report receptive syringe sharing within the past month at first interview (*X*^2^ = 7.49, *p* = 0.006). Furthermore, those with insufficient coverage recorded higher injecting risk scores on the BBV-TRAQ-SV scale at both interviews, a difference that was significant at most recent interview (*p* = 0.022), but not first.

At first interview, participants with current HCV infection were more likely to report insufficient coverage (*X*^2^ = 8.78, *p* = 0.012). This finding was confirmed in regression analysis, which identified greater odds of insufficient coverage for those with a current HCV infection (adjusted odds ratio (AOR)) = 4.44 (95 % confidence intervals (CIs): 1.43, 13.73)). There was little difference in coverage between HCV status subgroups at most recent interview. Regression analysis also showed reduced odds of insufficient coverage for participants who reported most commonly acquiring syringes from NSPs (as opposed to pharmacies or informal sources) (AOR = 0.27 (95 % CIs: 0.09, 0.77)).

Full descriptive and regression results are presented in Table 1.

**Table 1:** Analysis of recent sufficient and insufficient coverage at first and most recent interview

	First interview <100 %, *n* (%)	First interview ≥100 %, *n* (%)	Chi-squared *p*-value	Most recent interview <100 %, *n* (%)	Most recent interview ≥100 %	Chi-squared *p*-value	AOR^a^ at first interview, AOR (95 % CI)	AOR *p*-value
Sex								
*Female*	27 (36 %)	84 (39 %)	0.673	23 (32 %)	81 (38 %)	0.393	1	
*Male*	47 (64 %)	130 (61 %)		47 (68 %)	132 (62 %)		1.37 (0.61, 3.09)	0.442
Indigenous status								
*No*	66 (92 %)	200 (93 %)	0.606	62 (91 %)	202 (95 %)	0.270	1	
*Yes*	6 (8 %)	14 (7 %)		6 (9 %)	11 (5 %)		1.04 (0.26, 4.20)	0.956
WHO definition of youth								
*≤24 years*	11 (15 %)	41 (19 %)	0.408	3 (4 %)	9 (4 %)	0.983	1	
*>24 years*	63 (85 %)	173 (81 %)		67 (96 %)	204 (96 %)		0.77 (0.23, 2.51)	0.661
Highest level of education								
*<yr10*	23 (31 %)	63 (29 %)	0.515	15 (22 %)	59 (28 %)	0.620	1	
*Year 10–11*	26 (36 %)	92 (43 %)		26 (38 %)	74 (35 %)		0.56 (0.23, 1.39)	0.210
*Year 12/higher educ/trade*	24 (33 %)	59 (28 %)		28 (40 %)	80 (37 %)		1.61 (0.63, 4.13)	0.324
Employment status								
*No*	63 (85 %)	175 (82 %)	0.511	56 (80 %)	168 (79 %)	0.840	1	
*Yes*	11 (15 %)	39 (18 %)		14 (20 %)	45 (21 %)		0.94 (0.29, 3.01)	0.919
Weekly income								
*<$400*	52 (70 %)	144 (67 %)	0.635	35 (50 %)	113 (53 %)	0.657	1	
*≥$400*	22 (30 %)	70 (33 %)		35 (50 %)	100 (47 %)		0.97 (0.40, 2.35)	0.943
Stable accommodation								
*No*	16 (22 %)	31 (14 %)	0.152	17 (25 %)	34 (16 %)	0.104	1	
*Yes*	58 (78 %)	183 (86 %)		52 (75 %)	179 (84 %)		1.03 (0.39, 2.71)	0.956
Country of birth								
*Other*	17 (24 %)	40 (19 %)	0.366	18 (26 %)	36 (17 %)	0.081	1	
*Australia*	55 (76 %)	174 (81 %)		50 (74 %)	177 (83 %)		0.96 (0.37, 2.46)	0.934
Injecting career								
*<13 years*	32 (45 %)	96 (45 %)	0.951	31 (46 %)	45 (21 %)	<0.001*	1	
*≥13 years*	40 (55 %)	118 (55 %)		37 (54 %)	168 (79 %)		0.65 (0.28, 1.53)	0.325
Heroin injection (past month)								
*No*	9 (12 %)	38 (18 %)	0.262	10 (14 %)	39 (18 %)	0.440	1	
*Yes*	65 (88 %)	176 (82 %)		60 (86 %)	174 (82 %)		2.30 (0.70, 7.56)	0.171
Methamphetamine injection (past month)								
No	40 (54 %)	141 (66 %)	0.069	20 (29 %)	118 (55 %)	<0.001*	1	
*Yes*	34 (46 %)	73 (34 %)		50 (71 %)	95 (45 %)		1.94 (0.86, 4.39)	0.112
Hazardous drinking scale score (8 point cut-off)								
*abstinent*	24 (33 %)	71 (33 %)	0.588	28 (40 %)	85 (40 %)	0.675	1	
*<8 points*	27 (37 %)	90 (42 %)		23 (31 %)	77 (36 %)		0.82 (0.34, 1.98)	0.656
*≥8 points*	22 (30 %)	52 (25 %)		20 (29 %)	51 (24 %)		1.66 (0.63, 4.36)	0.304
Current OST prescription								
*No*	35 (47 %)	86 (40 %)	0.199	45 (64 %)	86 (40 %)	0.001*	1	
*Yes*	29 (53 %)	127 (60 %)		25 (36 %)	127 (60 %)		1.02 (0.49, 2.11)	0.952
BBV-TRAQ-SV injecting risk scale score (continuous measure)								
*Mean*	8.91	6.04	0.083	9.64	5.58	0.022*	1.01 (0.98, 1.03)	0.652
Receptive sharing (past month)								
*No*	58 (78 %)	193 (91 %)	0.006*	57 (81 %)	191 (90 %)	0.054	1	
*Yes*	16 (22 %)	20 (9 %)		13 (19 %)	21 (10 %)		1.01 (0.31, 3.36)	0.982
Injecting others (past month)								
*No*	56 (76 %)	173 (81 %)	0.343	60 (86 %)	184 (86 %)	0.888	1	
*Yes*	18 (24 %)	41 (19 %)		10 (14 %)	29 (14 %)		1.20 (0.47, 3.05)	0.699
Injected by others (past month)								
*No*	66 (89 %)	186 (87 %)	0.610	68 (97 %)	192 (90 %)	0.063	1	
*Yes*	8 (11 %)	28 (13 %)		2 (3 %)	21 (10 %)		0.32 (0.69, 1.50)	0.148
Injecting more than usual (past six months)								
*No*	45 (61 %)	133 (62 %)	0.838	38 (54 %)	144 (68 %)	0.039*	1	
*Yes*	29 (39 %)	81 (38 %)		32 (46 %)	68 (32 %)		1.27 (0.59, 2.76)	0.541
Solitary injecting >80 % of the time								
*No*	48 (65 %)	152 (71 %)	0.321	52 (74 %)	154 (73 %)	0.788	1	
*Yes*	26 (35 %)	62 (29 %)		18 (26 %)	58 (27 %)		1.43 (0.64, 3.18)	0.384
Arrest (since last interview)								
*No*	28 (38 %)	109 (51 %)	0.048*	31 (45 %)	114 (54 %)	0.202	1	
*Yes*	46 (62 %)	104 (49 %)		38 (55 %)	98 (46 %)		1.53 (0.70, 3.34)	0.281
HCV serology status								
*Negative*	6 (10 %)	43 (24 %)	0.012*	6 (10 %)	19 (11 %)	0.607	1	
*Positive*	42 (70 %)	87 (49 %)		42 (68 %)	105 (61 %)		**4.44 (1.43, 13.73)**	0.010*
*Exposed*	12 (20 %)	47 (27 %)		14 (22 %)	59 (28 %)		1.66 (0.46, 6.01)	0.436
NSP as usual source of syringe acquisition (past month)								
*No*	12 (16 %)	20 (9 %)	0.105	20 (29 %)	31 (15 %)	0.008*	1	
*Yes*	62 (84 %)	194 (91 %)		50 (71 %)	182 (85 %)		**0.27 (0.09, 0.77)**	0.015*

Regression number of observations: 215; Prob(chi^2^): 0.12; R^2^: 0.14

*Indicates statistically significant result at the <0.05 alpha level (bold data)

^a^Adjusted Odds Ratio, adjusted for all variables in the table

### Coverage pattern group categorisation

Of participants with valid data for coverage pattern categorisation (*n* = 322), 162 (50 %) were consistently covered, 17 (5 %) were consistently uncovered and 143 (45 %) were inconsistently covered.

Median coverage across interviews for the total cohort was 150–167 %. The consistently covered group had greater median levels at every interview wave (214–250 %); the reverse was true for the consistently uncovered group, who experienced at least a 50 % shortfall in median coverage (45–50 %). Inconsistently covered participants recorded over 100 % median coverage (102–117 %).

**Fig. 2:**
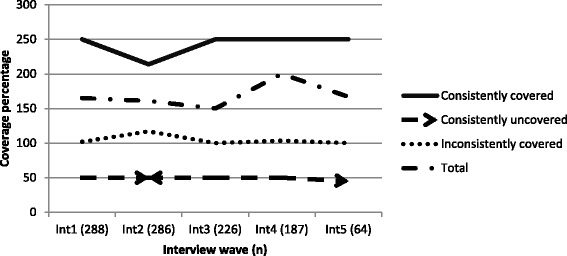
Median coverage percentage across interview waves by coverage pattern groups

Longitudinal median coverage data are presented in Fig. 2.

### Correlates of coverage pattern groups at first interview

Most exposure sub-groups were proportionally similar between coverage pattern groups. However, some significant differences were found.

Those consistently covered were significantly less likely to have receptively shared syringes within the past month (*X*^2^ = 9.58, *p* = 0.008) than the other coverage pattern groups. They were also significantly more likely to have injecting careers equal to or longer than 13 years (*X*^2^ = 6.58, *p* = 0.037) and current OST prescription (*X*^2^ = 12.60, *p* = 0.002).

In regression analysis, two significant results were found. Those with a current prescription of OST had decreased odds of being classified as inconsistently covered (AOR = 0.41 (95 % CIs: 0.22, 0.76)), whilst those with a current HCV infection had increased odds of being classified as inconsistently covered (AOR = 2.73 (95 % CIs: 1.12, 6.64)).

Full descriptive and regression results are presented in Table 2.

**Table 2:** Descriptive and logistic regression analysis of coverage pattern groups at first interview

	Consistently covered, *n* (%)	Consistently uncovered, *n* (%)	Inconsistently covered, *n* (%)	Chi squared *p*-value	AOR^a^ at first interview, AOR (95 % CI)	AOR *p*-value
Sex						
*Female*	67 (41 %)	6 (35 %)	49 (34 %)	0.433	1	
*Male*	95 (59 %)	11 (65 %)	94 (66 %)		1.43 (0.72, 2.83)	0.311
Indigenous status						
*No*	152 (94 %)	14 (88 %)	136 (96 %)	0.361	1	
*Yes*	10 (6 %)	2 (12 %)	6 (4 %)		0.33 (0.07, 1.62)	0.172
WHO definition of youth						
*≤24*	22 (14 %)	2 (12 %)	28 (19 %)	0.321	1	
*>24*	140 (86 %)	15 (88 %)	115 (81 %)		1.50 (0.18, 1.37)	0.177
Highest level of education						
*<yr10*	53 (33 %)	6 (35 %)	36 (25 %)	0.613	1	
*yr 10–11*	64 (39 %)	6 (35 %)	66 (47 %)		1.73 (0.82, 3.62)	0.148
*yr 12/higher educ/trade*	45 (28 %)	5 (30 %)	40 (28 %)		2.14 (0.92, 5.01)	0.078
Employment status						
*No*	125 (77 %)	15 (88 %)	118 (83 %)	0.348	1	
*Yes*	37 (23 %)	2 (12 %)	25 (17 %)		0.91 (0.37, 2.23)	0.838
Weekly income						
*<$400*	109 (68 %)	10 (59 %)	101 (71 %)	0.581	1	
*≥$400*	52 (32 %)	7 (41 %)	42 (29 %)		1.01 (0.48, 2.13)	0.976
Stable accommodation						
*No*	21 (13 %)	2 (12 %)	22 (15 %)	0.801	1	
*Yes*	141 (87 %)	15 (88 %)	121 (85 %)		1.03 (0.44, 2.41)	0.946
Country of birth						
*Other*	24 (15 %)	5 (31 %)	29 (20 %)	0.169	1	
*Australia*	138 (85 %)	11 (69 %)	113 (80 %)		0.93 (0.38, 2.25)	0.873
Injecting career						
*<13 years*	58 (36 %)	8 (50 %)	71 (50 %)	0.037*	1	
*≥13 years*	104 (64 %)	8 (50 %)	71 (50 %)		0.62 (0.30, 1.25)	0.181
Heroin injection (past month)						
*No*	41 (25 %)	2 (12 %)	24 (17 %)	0.120	1	
*Yes*	121 (75 %)	15 (88 %)	119 (83 %)		1.26 (0.47, 3.34)	0.645
Methamphetamine injection (past month)						
No	108 (67 %)	11 (65 %)	92 (64 %)	0.910	1	
Yes	54 (33 %)	6 (35 %)	51 (36 %)		0.93 (0.46, 1.87)	0.842
Hazardous drinking scale score (8 point cut-off)						
*abstinent*	53 (33 %)	5 (29 %)	41 (29 %)	0.827	1	
*<8 points*	69 (43 %)	9 (53 %)	62 (44 %)		1.17 (0.57, 2.40)	0.673
*≥8 points*	39 (24 %)	3 (18 %)	39 (27 %)		1.42 (0.59, 3.42)	0.437
Current OST prescription						
*No*	54 (33 %)	11 (65 %)	72 (50 %)	0.002*	1	
*Yes*	108 (67 %)	6 (35 %)	71 (50 %)		**0.41 (0.22, 0.76)**	0.005*
BBV-TRAQ-SV injecting risk scale score (continuous measure)						
*Mean*	6.03	9.88	5.96	0.293	0.97 (0.97, 1.02)	0.776
Receptive sharing (past month)						
*No*	150 (93 %)	12 (71 %)	124 (87 %)	0.008*	1	
*Yes*	11 (7 %)	5 (29 %)	19 (13 %)		1.10 (0.36, 3.35)	0.865
Injecting others (past month)						
*No*	137 (85 %)	13 (76 %)	119 (83 %)	0.686	1	
*Yes*	25 (15 %)	4 (24 %)	24 (17 %)		0.97 (0.40, 2.32)	0.945
Injected by others (past month)						
*No*	145 (90 %)	16 (94 %)	128 (90 %)	0.830	1	
*Yes*	17 (10 %)	1 (6 %)	15 (10 %)		1.66 (0.44, 6.23)	0.452
Injecting more than usual (past six months)						
*No*	107 (66 %)	12 (71 %)	88 (62 %)	0.625	1	
*Yes*	54 (34 %)	5 (29 %)	54 (38 %)		1.55 (0.80, 3.00)	0.191
Solitary injecting >80 % of the time						
*No*	101 (67 %)	8 (50 %)	96 (71 %)	0.240	1	
*Yes*	50 (33 %)	8 (50 %)	40 (29 %)		0.99 (0.50, 1.96)	0.985
Arrest (since last interview)						
*No*	89 (55 %)	6 (35 %)	64 (45 %)	0.090	1	
*Yes*	45 (45 %)	11 (65 %)	79 (55 %)		1.28 (0.68, 2.40)	0.439
HCV serology status						
*Negative*	27 (21 %)	2 (17 %)	16 (14 %)	0.451	1	
*Positive*	65 (50 %)	8 (66 %)	68 (61 %)		**2.73 (1.12, 6.64)**	0.027*
*Exposed*	37 (29 %)	2 (17 %)	28 (25 %)		2.31 (0.87, 6.13)	0.093
NSP as usual source of syringe acquisition (past month)						
*No*	14 (9 %)	4 (25 %)	18 (13 %)	0.149	1	
*Yes*	137 (91 %)	12 (75 %)	120 (87 %)		0.96 (0.36, 2.54)	0.933

Regression number of observations: 212; Prob(chi2): 0.25; R^2^: 0.10

*Indicates statistically significant result at the <0.05 alpha level (bold data)

^a^Adjusted Odds Ratio, adjusted for all variables in the table

## Discussion

We conducted longitudinal analysis of individual syringe coverage to address the gap noted in previous research [11] and to better understand the characteristics and predictors of coverage.

We found substantial levels of insufficient coverage. Across interview waves, the percentage of participants experiencing insufficient coverage was between 22–36 %, a finding that accords with previous Australian research [2, 12]. The fact that, at any time point, between a fifth to a third of the sample have “uncovered” injecting episodes is of serious concern, particularly considering that insufficiently covered participants in this study had a greater tendency to report receptive sharing of syringes, another finding that confirms past research [2, 11].

Analysis of longitudinal coverage patterns showed that most participants were consistently able to achieve sufficient coverage across interviews. The levels of insufficient coverage seen at each interview wave were driven then, not by those consistently uncovered but by those who fluctuated between states of coverage over time. This oscillating group should be the focus of interventions designed to reduce insufficient coverage. That so many participants were able to cover themselves at some time points but not at others, suggests a relationship between individual coverage and temporal context, rather than a consistent pattern of deficient coverage.

Cross-sectional analysis revealed that NSP access was associated with higher levels of coverage. This finding is plausible and highlights the advantages of harm reduction services from which PWID can reliably acquire syringes for free. These services overcome the inherent barriers of commercial sources (such as pharmacies) and potentially inconsistent or unreliable sources (such as friends and partners).

The association between insufficient coverage and current HCV infection (RNA positive) was strong. Previous research has shown that knowledge of HCV negativity can moderate injecting risk behaviours, such as receptively sharing syringes or injecting equipment [24]. A similar association between HCV status and coverage may be hypothesised, whereby a current HCV infection confers a consequent negligence with regards to sufficient syringe acquisition. Conversely, the shortfall in coverage might be a driver of HCV transmission.

We identified a persistent association between coverage and OST prescription. Cross-sectionally, those with a current OST prescription had significantly higher proportions of sufficient coverage (an outcome Bluthenthal et al. [11] also identified), and longitudinally, current OST prescription was significantly associated with being in the “consistently covered” group. We suspect that the key driver here is the efficacy of OST in reducing opiate use [20, 25]. Receipt of OST has been shown to reduce the risk of HCV incidence amongst Australian heroin injecting PWID almost five-fold [26], whilst internationally, combined OST prescription with sufficient individual-level coverage (termed “full harm reduction”) has been associated with an almost 80 % decrease in the risk of HCV acquisition [27]. The role of OST prescription in reducing HCV transmission is reflective of a reduction in injecting risk. Subsequently, the expansion of OST provision may play an important role in increasing coverage levels. Victorian OST services, however, are currently hampered by insufficient prescribers and inefficiencies in service co-ordination [28]. Increasing the numbers of PWID in receipt of OST would require strategies to overcome these barriers.

Though Australia’s harm reduction provision is comprehensive, with at least one source of syringe distribution per 30 PWID [16], the proportions of insufficient coverage in this and similar Australian research [2, 12, 17] indicate ongoing shortfalls. One explanation is that the PWID population is dynamic and diverse. The variance in individual coverage is undoubtedly due to more factors than we’ve captured here (as evidenced by the regression model’s low R^2^ value). To appropriately account for this diversity, harm reduction services must be adaptive and flexible. Consequently, the acquisition of sterile syringes should be facilitated as much as possible by expanding hours of NSP operation and implementing novel methods of syringe distribution (such as syringe vending machines, which are not widely available in Melbourne) [29–31]. NSPs are an efficacious, cost-effective means of limiting disease spread [14, 32, 33], and recent modelling suggests increases in service coverage would decrease BBV prevalence [34, 35].

Finally, research on individual coverage levels highlight the inadequacy of population-level measurements (such as the WHO measure). Though logistically difficult to determine, individual-level measurements capture the micro-details of coverage that are often diluted in population-wide averages. For example, at first interview in our cohort, 14,525 syringes were reportedly acquired by 338 currently injecting participants within the two weeks before interview, or an average of 43 syringes per person. If this average was multiplied by 26 to extrapolate to the total weeks in the year, this equals 1118 syringes per PWID, nearly six times the WHO recommendation for syringe distribution to curtail HIV spread [6]. However, it is clear that in aggregate, the PWID who cover their injecting episodes mask those who do not, and those who do not cover themselves are at most risk.

### Limitations

To measure individual levels of coverage, separate parameters are required, all prone to reporting bias. Such a limitation is an unavoidable element of this field of study [11, 12]. However, PWID recall reliability has been demonstrated [36], and we chose the past two weeks as the recall period for the questions to minimise recall bias.

Recent research has shown that many PWID exploit Australia’s unlimited dispensation policy and stockpile syringes for future use [17], meaning that participants who reported no past two-week syringe acquisition may still have been sufficiently covered. These findings came after MIX survey development and we were unable to account for stockpiling in our dataset. However, McCormack et al. found that the inclusion of a stockpiling question decreased levels of insufficient coverage (also using Bluthenthal et al.’s measure) by only eight percentage points (24 to 16 %) across their sample [17], so we are confident in the patterns we observed.

A substantial amount of coverage data was missing from our dataset. Approximately 45 % of our observations lacked coverage data, mostly (72 %) due to past week injection abstinence. However, the remaining 28 % of missing data was due to no reported syringe acquisition within the past two weeks and, with many of these participants also reporting injecting (sometimes in significant frequencies), syringe stockpiling was probably occurring. Therefore, we restricted analysis to those participants reporting both injecting and concurrent syringe acquisition.

Finally, our participants were recruited from a population with unknown parameters, limiting the generalisability of our findings [37].

## Conclusion

We explored individual needle and syringe coverage longitudinally. We replicated previous Australian research and found substantial insufficient coverage amongst our group. This coverage shortfall is driven mainly by participants who cover themselves intermittently, suggesting the influence of temporal factors. Statistical analysis showed the protective effects of current OST prescription and NSPs as the main source of syringe acquisition, and an increased risk for those currently infected with HCV. An increase in OST coverage would potentially see a concurrent increase in syringe coverage, whilst more generally, to ensure PWID have every opportunity to avoid BBV infections and other injecting-related problems, the best response is the general expansion of NSP services.

## Additional files

Additional file 1: Appendix 1. Demographic comparison between total and amended MIX samples at first interview. Comparative statistics between the full MIX cohort and the amended cohort used in this analysis. (DOCX 14 kb) Additional file 2: Amended MIX questionnaire for longitudinal coverage analysis. Selected questions from the MIX questionnaire, relevant to the analysis within this article. Variables are presented in their original state, prior to data cleaning. (DOCX 22 kb)

## Abbreviations

AOR: Adjusted odds ratio
BBV: Blood-borne virus
95 % CIs: 95 % Confidence intervals
HCV: Hepatitis C virus
IQR: Interquartile range
MIX: Melbourne injecting drug user cohort study
NSP: Needle and syringe program
OST: Opioid substitution therapy
PWID: People/person who injects drugs
WHO: World Health Organisation
