# Supplementary material for: Individual-level needle and syringe coverage in Melbourne, Australia: a longitudinal, descriptive analysis
Source: BMC Health Serv Res. 2016 Aug 19;16:411. doi: 10.1186/s12913-016-1668-z (PMC4992312; doi:10.1186/s12913-016-1668-z)
Supplement: Additional file 1: — Appendix 1. Demographic comparison between total and amended MIX samples at first interview. Comparative statistics between the full MIX cohort and the amended cohort used in this analysis. (DOCX 14 kb) [file 12913_2016_1668_MOESM1_ESM.docx]

**Appendix 1: Demographic comparison between total and amended MIX samples at first interview**

| **Characteristic** | **Total sample (N=757)** | **Amended sample (N=502)** |
| --- | --- | --- |
| Sex (male/female) | 502 (66%) / 255 (34%) | 321 (64%) / 181 (36%) |
| Age (mean, range) | 28, 17-52 | 30, 19-52 |
| Employment (yes/no) | 114 (15%) / 640 (85%) | 109 (22%) / 393 (78%) |
| Accommodation (stable/unstable) | 610 (81%) / 141 (19%) | 427 (85%) / 75 (15%) |
| Country of birth (Aust/other) | 596 (79%) / 159 (21%) | 410 (82%) / 90 (18%) |
| ATSI (yes/no) | 45 (6%) / 710 (94%) | 26 (5%) / 474 (95%) |
| Age of initiation (mean, range) | 17, 8-32 | 17, 10-32 |
| Drug most injected in past month (heroin/other) | 563 (76%) / 176 (24%) | 304 (73%), 112 (27%) |
| Currently prescribed OST (yes/no) | 285 (38%) / 472 (62%) | 294 (59%), 208 (41%) |
| Past week injection frequency (mean, range) | 8, 0-139 | 6, 0-65 |
