# Supplementary material for: Individual-level needle and syringe coverage in Melbourne, Australia: a longitudinal, descriptive analysis
Source: BMC Health Serv Res. 2016 Aug 19;16:411. doi: 10.1186/s12913-016-1668-z (PMC4992312; doi:10.1186/s12913-016-1668-z)
Supplement: Additional file 2: — Amended MIX questionnaire for longitudinal coverage analysis. Selected questions from the MIX questionnaire, relevant to the analysis within this article. Variables are presented in their original state, prior to data cleaning. (DOCX 22 kb) [file 12913_2016_1668_MOESM2_ESM.docx]

**Amended MIX questionnaire for longitudinal coverage analysis**

**Baseline demographic questions**

A3. Enter participant's sex 1 Male

0 Female

9 Not Applicable

A4. What's your date of birth? __ __ / __ __ / __ __ __ __ dd / mm / yyyy

2097 Don't Know (Year)

2098 Refuse to Answer(Year)

2099 Not Applicable (Year)

A5. What is the highest level of education that you have completed? (Choose one)

__ __ Dont know

__ __ None

__ __ Primary School

__ __ Year 7-9

__ __ Year 10-11

__ __ Year 12/completed secondary school

__ __ Tertiary

__ __ Diploma/associate diploma/advanced diploma

__ __ trade/technical qualification or TAFE qualification at

__ __ Other

__ __ Don't Know

__ __ Refuse to Answer

__ __ Not Applicable

A19. What was your country of birth? (Choose one) 00 Australia

01 United Kingdom

02 New Zealand

03 Italy

04 Vietnam

05 China

06 Greece

49 Other

97 Don't Know

98 Refuse to Answer

99 Not Applicable

A23. Are you of Aboriginal and/or Torres Strait Islander origin? (Choose one)

1 No

2 Yes, Aboriginal

3 Yes, Torres Strait Islander

4 Yes, Aboriginal and Torres Strait Is.

7 Don't Know

8 Refuse to Answer

9 Not Applicable

B1. How old were you when you first injected a drug? __ __ years

997 Don't Know

998 Refuse to Answer

999 Not Applicable

**Follow-up questions**

A4. How are you employed at the moment?
*(Select on response only)* (Choose one) 01 Not employed

02 Full time

03 Part time/casual

04 Full time student

05 Home duties

06 Student/employed

49 Yes, other

97 Don't Know

98 Refuse to Answer

99 Not Applicable

A14. Which of the following would represent your **average weekly income** in the past six months (before tax) from all sources (including illicit)? (Choose one)

01 $2,000

02 $1,600 - $1,999

03 $1,300 - $1,599

04 $1,000 - $1,299

05 $800 - $999

06 $600 - $799

07 $400 - $599

08 $250 - $399

09 $150 - $249

10 $1 - $149

11 Nil income

12 Negative income

97 Don't Know

98 Refuse to Answer

99 Not Applicable

A17. What type of accommodation do you *currently* live in? (Choose one)

01 owner occupied property

02 Rental property (private)

03 Rental property (public)

04 Boarding (eg paying for accommodation with family/friends)

05 Boarding house

06 Institution

07 Squat

08 homeless/street

49 other

97 Don't Know

98 Refuse to Answer

99 Not Applicable

B160. Over the last month, about how often did you use alone? *(ask for percentage or fraction, but enter percentage)*

__ __ __

997 Don't Know

998 Refuse to Answer

999 Not Applicable

D38. In the past 6 months, have there been periods of a month or more when you've injected more than usual?

1 Yes

0 No

7 Don't Know

8 Refuse to Answer

9 Not Applicable

E95. Have you been arrested **in the last 12 months**? 1 Yes

0 No

7 Don't Know

8 Refuse to Answer

9 Not Applicable

**Measuring past week injecting frequency.**

B8. How many times have you injected **heroin** in the last week? __ __

97 Don't Know

98 Refuse to Answer

99 Not Applicable

B14. How many times have you injected **methadone that was not prescribed** in the last week?

__ __

97 Don't Know

98 Refuse to Answer

99 Not Applicable

B20. How many times have you injected **buprenorphine that was not prescribed** in the last week?

__ __

97 Don't Know

98 Refuse to Answer

99 Not Applicable

B28. How many times have you injected **suboxone that was not prescribed** in the last week?

__ __

97 Don't Know

98 Refuse to Answer

99 Not Applicable

B35. How many times have you injected **morphine that was not prescribed** in the last week?

__ __

97 Don't Know

98 Refuse to Answer

99 Not Applicable

B41. How many times have you injected **oxycodone that was not prescribed** in the last week?

__ __

97 Don't Know

98 Refuse to Answer

99 Not Applicable

B47. How many times have you injected **other opiates that were not prescribed** in the last week?

__ __

97 Don't Know

98 Refuse to Answer

99 Not Applicable

B53. How many times have you injected **speed powder** in the last week? __ __

97 Don't Know

98 Refuse to Answer

99 Not Applicable

B59. How many times have you injected **base/point/wax** in the last week? __ __

97 Don't Know

98 Refuse to Answer

99 Not Applicable

B65. How many times have you injected **ice/crystal/shabu** in the last week? __ __

97 Don't Know

98 Refuse to Answer

99 Not Applicable

B71. How many times have you injected **prescription stimulants that were prescribed to you** in the last week? __ __

97 Don't Know

98 Refuse to Answer

99 Not Applicable

B77. How many times have you injected **prescription stimulants that were not prescribed to you** in the last week? __ __

97 Don't Know

98 Refuse to Answer

99 Not Applicable

B83. How many times have you injected **cocaine** in the last week? __ __

97 Don't Know

98 Refuse to Answer

99 Not Applicable

B91. How many times have you injected **hallucinogens** in the last week? __ __

97 Don't Know

98 Refuse to Answer

99 Not Applicable

B97. How many times have you injected **ecstasy** in the last week? __ __

97 Don't Know

98 Refuse to Answer

99 Not Applicable

B108. How many times have you injected **benzodiazepines** in the last week? __ __

97 Don't Know

98 Refuse to Answer

99 Not Applicable

B122. How many times have you injected **antipsychotics** in the last week? __ __

97 Don't Know

98 Refuse to Answer

99 Not Applicable

B133. How many times have you injected **antidepressants/mood stablisers** in the last week?

__ __

97 Don't Know

98 Refuse to Answer

99 Not Applicable

**Past fortnight syringe coverage**

B164. In the last month, where did you usually get your needles/syringes from? (Choose one)

1 needle and syringe program

2 chemist/pharmacy

3 partner/friends

4 dealer

5 dont know

6 other

7 Don't Know

8 Refuse to Answer

9 Not Applicable

B166. How many times in the last two weeks did you get needles and syringes?

__ __ __ __

9997 Don't Know

9998 Refuse to Answer

9999 Not Applicable

B167. In the last two weeks how many new syringes in total did you get?

__ __ __ __

9997 Don't Know

9998 Refuse to Answer

9999 Not Applicable

B166. In the last two weeks how many syringes did you give away or sell to others?

__ __ __ __

9997 Don't Know

9998 Refuse to Answer

9999 Not Applicable

**Current OST prescription**

If D2 is not equal to 1, then skip to D6.

D3. Are you currently on methadone maintenance treatment? 1 Yes

0 No

7 Don't Know

8 Refuse to Answer

9 Not Applicable

If D6 is not equal to 1, then skip to D10.

D7. Are you currently on buprenorphine maintenance treatment? 1 Yes

0 No

7 Don't Know

8 Refuse to Answer

9 Not Applicable

D11. Are you currently on suboxone maintenance treatment? 1 Yes

0 No

7 Don't Know

8 Refuse to Answer

9 Not Applicable

D16. Are you currently on naltrexone maintenance treatment? 1 Yes

0 No

7 Don't Know

8 Refuse to Answer

9 Not Applicable
